# Endometrial Receptivity: A Revisit to Functional Genomics Studies on Human Endometrium and Creation of HGEx-ERdb

**DOI:** 10.1371/journal.pone.0058419

**Published:** 2013-03-26

**Authors:** Sonali R. Bhagwat, Darshan S. Chandrashekar, Ruchi Kakar, Sravanthi Davuluri, Akhilesh K. Bajpai, Sumeet Nayak, Sumit Bhutada, Kshitish Acharya, Geetanjali Sachdeva

**Affiliations:** 1 Primate Biology Department, National Institute for Research in Reproductive Health, Mumbai, Maharashtra, India; 2 Institute of Bioinformatics and Applied Biotechnology, Bangalore, Karnataka, India; Emory University School of Medicine, United States of America

## Abstract

**Background:**

Endometrium acquires structural and functional competence for embryo implantation only during the receptive phase of menstrual cycle in fertile women. Sizeable data are available to indicate that this ability is acquired by modulation in the expression of several genes/gene products. However, there exists little consensus on the identity, number of expressed/not-detected genes and their pattern of expression (up or down regulation).

**Methods:**

Literature search was carried out to retrieve the data on endometrial expression of genes/proteins in various conditions. Data were compiled to generate a comprehensive database, Human Gene Expression Endometrial Receptivity database (HGEx-ERdb). The database was used to identify the Receptivity Associated Genes (RAGs) which display the similar pattern of expression across different investigations. Transcript levels of select RAGs encoding cell adhesion proteins were compared between two human endometrial epithelial cell lines; RL95-2 and HEC-1-A by quantitative real time polymerase chain reaction (q-RT-PCR). Further select RAGs were investigated for their expression in pre-receptive (n = 4) and receptive phase (n = 4) human endometrial tissues by immunohistochemical studies. JAr spheroid attachment assays were carried out to assess the functional significance of two RAGs.

**Results:**

HGEx-ERdb (http://resource.ibab.ac.in/HGEx-ERdb/) helped identification of 179 RAGs, of which 151 genes were consistently expressed and upregulated and 28 consistently not-detected and downregulated in receptive phase as compared to pre-receptive phase. q-RT-PCR confirmed significantly higher (p<0.005) expression of Thrombospondin1 (THBS1), CD36 and Mucin 16 transcripts, in RL95-2 as compared to HEC-1-A. Further, the pretreatment with antibodies against CD36 and COMP led to a reduction in the percentage of JAr spheroids attached to RL95-2. Immunohistochemical studies demonstrated significantly higher (p<0.05) expression of endometrial THBS1, Cartilage Oligomeric Matrix Protein (COMP) and CD36 in the receptive phase as compared to pre-receptive phase human endometrial tissues.

**Conclusion:**

HGEx-ERdb is a catalogue of 19,285 genes, reported for their expression in human endometrium. Further 179 genes were identified as the RAGs. Expression analysis of some RAGs validated the utility of approach employed in creation of HGEx-ERdb. Studies aimed towards defining the specific functions of RAGs and their potential networks may yield relevant information about the major ‘nodes’ which regulate endometrial receptivity.

## Introduction

Endometrium, the inner lining of the uterus, is receptive to the embryo only during a defined period in the menstrual cycle. This period called as the ‘receptive phase’ or the window of implantation, is marked by structural and functional maturation of endometrium [1]–[3]. In view of the molecular complexities involved in endometrial maturation, it is rightly believed that the events underlying the endometrial receptivity are handiworks of several genes/gene-products. The clinical relevance of endometrial receptivity has prompted several investigators to pursue studies on specific and global gene expression profiling of human endometrium.

In recent years, several microarray based investigations have been undertaken to identify the genes/proteins which are expressed in human endometrium during the receptive phase [4]–[11]. These investigations were conducted in different study cohorts, and employed different sampling strategies, study design and analysis tools. To our knowledge no major strides have been made to arrive at a consensus on the genes, identified for their differential expression in the human endometrium during the receptive phase, across different datasets. In the present study, we adopted a systematic approach of converging the existing data on endometrial gene signatures and then scoring all the genes for their expression status (detected/not detected) as well as for their expression pattern (up or down regulation) in the receptive phase across different datasets [12]. The premise was that the screening for the “commons” in different data sets, differing with regard to the sample size, study design, experimental strategies, analysis tools and ethnicity of the participants, may lead to identification of the genes with higher consensus on their association with endometrial receptivity. The effects of biological variations, which are not truly associated with endometrial receptivity, are expected to be eliminated by analysing the large sample size (pooled data sets).

In recent years, a few attempts have been made to assimilate the information on global gene expression profiling of human endometrial tissues as research resources in the form of either isolated reports or databases. Diaz-Gimeno et al. [13] employed Bioinformatics tools to create an Endometrial Receptivity Array (ERA). However, genes included in the array were selected from the data derived from a single study. Another *in silico* investigation derived the source data from 7 microarray based studies but focussed on the identification of transcription factors, which bind to the regulatory sequences of differentially expressed genes in the receptive phase endometrium [14]. Two databases also exist, Endometrial Database (http://www.endometrialdatabase.com) and SCCPIR Endometrium Database Resource (http://edr.research.bcm.edu/edr/ui-linksseams). The former is a catalogue of the investigations on natural and stimulated cycles; endometrial receptivity, implantation and endometrial disorders. It allows the queries by gene ID but does not provide structured data on the menstrual cycle phase specific gene signatures. SCCPIR (Specialized Cooperative Centers Program in Reproduction and Infertility Research) supports an online public database- Endometrial Database Resource (EDR), which provides information on the genes, reported to be expressed in the uterus in human, mouse, rat, cow, guinea pig, pig and sheep. EDR provides “gene specific” information in the context of uterus. However, it does not allow a user to retrieve “condition specific” gene signatures. The mammalian uterus database-MGEx-Udb [15] also lacks data on menstrual cycle phase specific gene signatures.

In the present study, existing data on the context specific endometrial expression profiling was manually curated and a database created. Further the database was screened to identify the genes which display a similar trend of expression during the receptive phase in different datasets. Select genes were validated for their expression pattern in two human endometrial epithelial cell lines, differing in their adherence to embryonic cells and thus partially simulating receptive and non-receptive endometrium. Select genes were also investigated for their protein expression in pre-receptive and receptive phase human endometrial tissue sections. Efforts were also made to assess the functional significance of two RAGs in the embryo-endometrial adhesion.

## Methods

### Data Compilation

**Figure 1:**
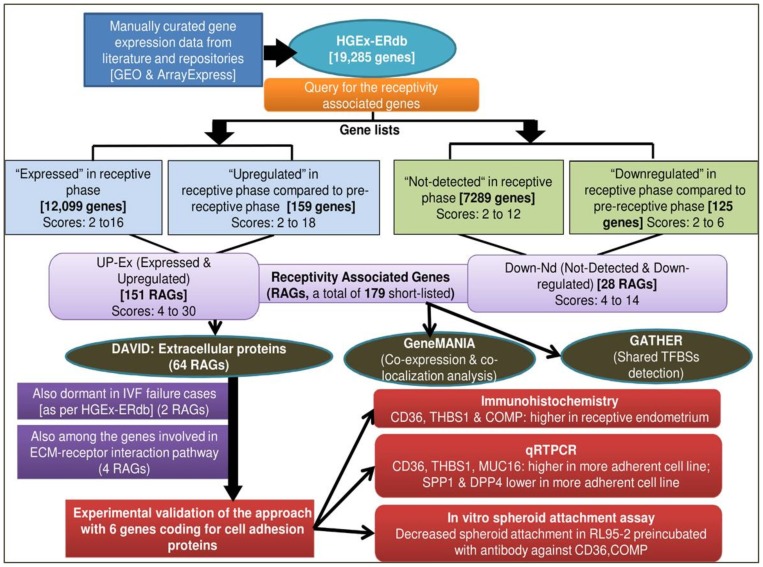
Strategies used for creation of HGEx-ERdb, *in silico* characterization of RAGs and experimental validation.

The strategies for creation of HGEx-ERdb are outlined in Figure 1. Published literature was searched extensively for the genes which are expressed in human endometrium at different stages of menstrual cycle, including those exposed to various conditions. For this, PubMed was searched with a carefully designed query set [(endometrium OR endometrial OR uterus OR uterine) AND (implants OR implantation OR implanting OR receptive OR receptivity OR fertil* OR “secretory phase” OR “proliferative phase” OR “ovulatory phase” OR non receptive OR IVF OR “in vitro fertilization” OR “embryo transfer”)] combined with keywords related to mass scale techniques. Full-text and supplementary materials of the relevant articles were screened for gene-lists (with at least 5 genes/proteins).

Gene Expression Omnibus (GEO), Array Express [http://www.ncbi.nlm.nih.gov/geo/, http://www.ebi.ac.uk/] and EDB, Endometrial database (www.endometrialdatabase.com) were screened for the gene signatures of human endometrium in native (different phases of the natural menstrual cycle) or pathological or experimental (*in vivo* or *in vitro* hormone/anti-hormone/gonadotropin stimulation) conditions. The lists of genes were collected along with information (datasets) about following parameters, in a specific format, and uploaded into a MySQL database:

- Ethnicity of the study participants.
- Treatment of the study participants, if any.
- Sample size.
- Experimental strategy (microarray, proteomics, qRT-PCR).
- Microarray platform and number of hybridizations in an experiment, in case, microarray technology is used.
- Validation experiments.
- Statistical methods.

### Creation of Human Gene Expression Endometrial Receptivity Database (HGEx-ERdb)

The database was created using the strategies described previously [15]. Briefly, perl based CGI script was used to create the interface for uploading of the gene lists and related information. The curated data were cross-checked by at least two investigators independently to eliminate the errors introduced during manual curation and entries. The gene related details (e.g. gene aliases, chromosomal location, potential promoter sequence [−1000 to +200 bp], transcript details were downloaded from NCBI, with the aid of NCBI E-utilities (http://eutils.ncbi.nlm.nih.gov/entrez/query/static/eutils_help.html). Protein related data were downloaded from UniProt (http://www.uniprot.org). Transcription start sites were retrieved from dbTSS (ftp://ftp.hgc.jp/pub/hgc/db/dbtss/) [16]. Ontology and protein interaction details were downloaded from Gene Ontology (ftp://ftp.geneontology.org/pub/go/) [17] and Biological General Repository for Interaction Datasets (BioGRID, http://thebiogrid.org/download.php) [18] databases, respectively. MySQL Relational Database Management System (RDBMS) was used for storing all the data.

### Derivation of Reliability Scores

Consistency of genes in terms of their expression status (expressed vs. not-detected), across various datasets, was assessed using a computational method described earlier [12] with some modifications. Genes with similar expression status (expressed vs not-detected), across different data sets, received higher score for that expression status in a specific condition. A gene received lower score if there were disagreements between different data sets or if there were less number of studies reporting this gene. In addition to counting the present (for expression)/absent (for not detected) calls for each gene, the modified program counted up and down-regulated incidents. Thus, the database has an ability to display a reliability score for the “expressed” and “not detected” status in specific conditions, and also for the pattern of expression (up or down regulation) across the conditions to be compared, such as pre-receptive vs receptive or mid- proliferative vs mid-secretory phases.

### Derivation of Receptivity Associated Genes (RAGs) and their *in-silico* Analysis

HGEx-ERdb was queried to identify the genes which display differential expression in human endometrium during the receptive phase. These genes were designated as Receptivity Associated Genes (RAGs) (Figure 1). For identification of RAGs, the database was queried for the genelists of receptive and pre-receptive endometrium of normal healthy women only. Genelists derived from the studies on the patients with any gynaecological disorder (endometriosis, fibroids, polycystic ovarian disorder, stimulated cycles) were not considered. On querying, the database displayed individual scores of each gene (reported to be expressed in a specific condition for example receptive or pre- receptive phase) for the expression status (expressed vs not detected) and also for the expression pattern (upregulated vs downregulated). Two sets of RAGs were identified a) Up-Ex i.e. consistently expressed (Ex) and upregulated (Up) b) Down-Nd i.e. consistently not-detected (Nd) and down regulated (Down); in the receptive phase compared to the pre-receptive phase. For each Up-Ex RAG gene, the scores for expressed and up-regulated status were added, to get a cumulative score. Similarly, scores for not-detected status and down-regulated status were added, to get a cumulative score for the Down-Nd RAGs. The cumulative reliability score indicated its expression pattern across multiple genelists. A gene was assigned a score of two if found expressed/upregulated (or not detected/downregulated) in one dataset. For example, the Up-Ex gene SPP1 has a cumulative score of 30 (up-regulation score of 18 and a score of 12 for the expressed status) while GPX3 had a cumulative score of 26 (14 for the upregulation and 12 for the expressed status). The reliability score for the expressed status in the receptive phase for both the top scorers is 12, which means that the gene was found to be expressed in at least 6 studies. Similarly a score of 18 for the upregulation indicates that SPP1 was found to be upregulated during the receptive phase in at least 9 studies. This scoring strategy enabled identification of the Receptivity Associated Genes (RAGs) with higher reliability.

The RAGs were analysed for their biological process, molecular function, cellular location using databases such as the Database for Annotation, Visualization and Integrated Discovery (DAVID) [19], GeneMANIA [20] and Gene Ontology (GO) [17]. Analysis for the transcription factor binding sites was carried out using Gene Annotation Tool to Help Explain Relationships (GATHER) [21].

### Experimental validation of the selected RAGs

Five RAGs encoding cell adhesion proteins, which have not been previously investigated for their association with endometrial receptivity, were further investigated. Their differential expression was validated by q RT-PCR in endometrial epithelial cell lines RL95-2 (more adhesive to embryonic cells) and HEC-1-A (less adhesive to embryonic cells) [22]. Three genes were selected for validation by immunolocalization of their respective protein products in the pre-receptive and receptive phase human endometrial tissues. Two of these RAGs were assessed for their potential role in embryo adhesion by *in vitro* spheroid attachment assays.

### Antibodies

Antibody (mouse monoclonal) against human thrombospondin1 (TSP1) was procured from Sigma Aldrich, while polyclonal antibodies against human CD36 and COMP (Cartilage Oligomeric Matrix Protein) were procured from Epitomics (Burlingame, CA, USA). Antibodies against CD36 were directed against the extracellular region of protein. Secondary antibodies for immunohistochemistry were purchased from Vector Laboratories (Burlingame, CA, USA). Secondary antibody conjugated to Alexa flour 488 for immunofluorescence was obtained from Invitrogen (Dorset, UK). Rabbit and mouse IgG were procured from Millipore (Billerica, MA, USA).

### Cell line maintenance

Two human endometrial epithelial cell lines {RL95-2 (CRL-1671), HEC-1-A (HTB-112)} and a human trophoblastic cell line- JAr (HTB-144) were obtained from the American Type Culture Collection (ATCC). RL95-2 was maintained in a 1∶1 mixture of Dulbecco's Modified Eagle's medium (Sigma-Aldrich) and nutrient mixture F-12 containing 15 mM HEPES, L-glutamine, 1.2 g/L sodium bicarbonate and 5.0 mg/L insulin. HEC-1-A was maintained in McCoy's 5A modified medium (Sigma-Aldrich,) with 10 mM HEPES, L-glutamine, and 2.2 g/L sodium bicarbonate. JAr cells were grown in RPMI-1640 (Sigma-Aldrich) containing 1 mM sodium pyruvate 10 mM HEPES and 2.5 g D-Glucose/L (Sigma-Aldrich). Media were supplemented with 10% fetal bovine serum (Invitrogen, Carlsbad, CA, USA) and 1% penicillin streptomycin mixture (1∶1). Cells were grown at 37°C in water-saturated air containing 5% CO_2._ Spheroid attachment assays were carried out at different time points to confirm the differential adhesiveness of RL95-2 and HEC-1-A cell lines (Figure S1) as described below.

### Spheroid attachment assay

JAr cells (2.5×10^6)^ per 6 ml RPMI medium were agitated at 37°C in 5% CO_2_ on a rotator shaker at 110 rpm for 24 hrs [23], [24]. To distinguish JAr spheroids from RL95-2 cells, JAr spheroids were labeled with the membrane-permeable fluorescent dye CMFDA, 5-Chloromethylfluorescein Diacetate (Invitrogen, Dorset, UK). Spheroids were gently delivered onto a confluent monolayer of RL95-2 cells grown in 24-wells culture plate (Nunc, NY, USA). The co-culture was incubated at 37°C for 2 hours. Unattached spheroids were removed by centrifuging the plate at 10 g for 5 min with cover slips turned upside down. The medium containing unattached spheroids was removed. Attached spheroids were counted after removing the media. Percent attached spheroids were calculated by determining the fraction of attached spheroids from the total number of spheroids added. For antibody blocking experiments [25], RL95-2 cells were seeded at a density of 7.5×10^5^ cells per well. Next day, cells were incubated with antibodies against CD36/COMP or rabbit IgG at a concentration of 5–7.5 µg/ml for 2 hrs at 37°C. This was followed by washing the cells with media to remove unbound antibodies. CD36/COMP antibody or rabbit IgG treated RL95-2 cells were then checked for their ability to bind with JAr spheroids as mentioned above.

### Immunofluorescence studies

RL95-2 and HEC-1-A cells (approximately 5×10^5^) were seeded on coverslips in a 24 well plate. Next day, the cells were washed with PBS and fixed with 3.7% paraformaldehyde for 25 min at RT. The fixative was removed by washing the cells twice with PBS and blocking was done subsequently with 0.1% BSA for 1 hr. After a PBS wash, the cells were incubated with primary antibody CD36 (0.05 µg/µl) and COMP (0.02 µg/µl) overnight at 4°C. Next day, after a PBS wash, cells were incubated with Alexa 488 conjugated secondary antibody (0.02 µg/µl) for 11/2 hrs at 37°C. Cells were washed once with PBS and incubated with 4′, 6-diamidino-2-phenylindole (DAPI) (Roche, Penzberg, Germany) for 20 min. The coverslips were mounted on glass slides and images were taken using confocal microscope (Karl Zeiss LSM 510 Meta, Germany).

### Human endometrial sample collection

#### Ethics Statement

Endometrial tissues were collected from healthy regularly cycling women after the approval of the NIRRH Ethics Committee for Clinical Studies. The participants of the study number 140/2007 provided their written consent according to the procedure approved by the committee. Women of reproductive age (21–35 years) with a history of regular, monthly menses, at least one live birth and with no pelvic pathologies were enrolled in the study. Women using any hormonal contraceptive methods and women with history of systemic diseases like tuberculosis, diabetes, hypertension or gynecological diseases like endometriosis, adenomyosis, endometrial polyps, genital malignancies, luteal phase defects were excluded. Sections of pre-receptive (collected on day 2 post-ovulation, n = 4) and receptive (collected on day 6 post-ovulation, n = 4) endometrial tissues were used in the study.

Ovulation was monitored by serial ultrasonography (USG) to ascertain the follicular collapse. The first USG was done on day 6 or day 7 of the menstrual cycle, depending on length of the last menstrual cycle, the second USG on day 8 or day 9 and then daily until the follicular rupture was observed. Endometrial tissues were collected on day 2 and day 6 following the follicular rupture and categorized as pre-receptive and receptive samples respectively. The tissue was then retrieved from the probet head into a petri plate containing saline and washed free of blood contamination. The tissue was fixed in 10% formalin in PBS for 24 hrs, and transferred to 70% ethanol for 24 hrs, followed by dehydration in the ascending grades of ethanol for 1 hr each. The tissue was next transferred to a mixture of 50% ethanol and 50% xylene for 1 hr and then to 100% xylene for 15 min or till the tissue became clear. The tissue was then transferred to paraffin wax and incubated at 56°C for 2 hrs and then 37°C overnight. Blocks were prepared and sections of 5 µ were cut for immunohistochemical experiments within six months of their preparation.

### Quantitative Reverse Transcription PCR

Total RNA were extracted from RL95-2 and HEC-1-A growing at three different passage numbers, using trizol method as described previously [26]. In brief, the cells (1×10^6^) were homogenized in 1.0 ml Trizol Reagent (Invitrogen, Carlsbad, CA, USA), followed by addition of 0.2 ml of chloroform and centrifugation at 12,000 rpm for 15–20 min at 4°C. To the aqueous phase, isopropanol (0.5 ml/ml trizol) was added and after incubation at RT for 20 min, centrifugation was done at 12,000 rpm for 30 min at 4°C. The pellet was washed with 75% ethanol, dried and dissolved in 30 μl diethypyrocarbonate (DEPC)-treated H_2_O. RNA samples were treated with RNase-free DNase (2 U/μl) at 37°C for 30 min. RNA samples were re-extracted with trizol to remove DNase and dissolved in RNase free water. RNA samples were stored at −70°C till used further.

Total RNA samples were converted to cDNA using HIGH PRIME cDNA synthesis kit (Applied Biosystems, Carlsbad, CA, USA). One microgram of RNA was reverse transcribed using random primers, reverse transcriptase buffer, dNTP mix, MultiScribeTM reverse transcriptase and RNase inhibitor. The reactions were then incubated at 25°C for 10 min, 37°C for 120 min followed by 85°C for 5 sec and then stored at −20°C.

Taqman gene expression assays for the gene of interest (labelled with 6 carboxy fluorescein or FAM dye) and housekeeping gene- *18S* rRNA (labelled with VIC dye- patented by Applied Biosystems) were obtained from Invitrogen. The biplex reaction containing 1 µl of diluted cDNA (0.2 µg), 1X primer probes for the gene of interest and the housekeeping gene, 1X universal PCR master mix in the 10 μl reaction volume was dispensed per well in the 96 well optical plate and amplified using 7900 HT Real Time PCR System (Applied Biosystems) for 40 cycles, each with the following parameters: denaturation at 50°C for 15 secs, and annealing and extension at 60°C for 1 min each. Real time PCRs were carried out in triplicates for each sample.

Relative quantity (RQ) of the transcripts was determined using RQ Manager software (Applied Biosystems). Relative fold change or relative expression was calculated by the delta delta Ct method. Delta Ct is the Ct value for the sample (control/experimental) normalized to the endogenous housekeeping gene (18S rRNA). Delta delta Ct was calculated by subtracting delta Ct of the calibrator or control sample from that of the experimental sample. Relative fold change or relative expression (RE) was calculated using the formula: RE = 2? [-(Experimental – Control ΔΔCt)] Values were expressed as RE ± SEM. For MUC16, CD36 and TSP1, HEC-1-A was considered as the control sample and for SPP1 and DPP4, RL95-2 was considered as the control sample.

### Immunohistochemical localization

Endometrial sections of 5 µ thickness were deparaffinised in xylene and rehydrated through descending grades of methanol. Endogenous peroxidase activity was quenched by treating the sections with 0.3% H_2_O_2_ in methanol for 30 min. For localization of THBS1, CD36 and COMP, the sections were blocked with 1% horse or goat serum in phosphate buffered saline (PBS) for 1 hr. and then incubated with the respective primary antibodies, diluted at 0.2 µg/ml for TSP1 and at 0.25 µg/ml for CD36 and COMP for 16 hrs at 4°C. In the negative controls, rabbit and mouse IgGs replaced respective primary antibodies. Sections were washed twice in PBS and incubated with 1∶100 dilution of respective secondary biotinylated antibodies (Vector Laboratories, Burlingame, CA, USA) prepared in blocking solution for 2 hrs at RT. As per the manufacturer's instructions, solution A (avidin) and solution B (biotinylated horseradish peroxidase) were diluted 50 times in PBS. The sections were incubated in avidin-biotin-horseradish peroxidase complex (Vector Laboratories) for 30 min followed by addition of 1 mg/ml diaminobenzidene (Sigma-Aldrich,) prepared in 0.001% H_2_O_2_ in PBS for 10 min. The immunostained sections were counterstained with hematoxylin and then gradually dehydrated, cleared in xylene and mounted in DPX (Distyrene Plasticizer and Xylene).

The staining intensities for immunoreactive antigens in the endometrial epithelium and stroma were determined using the image analysis software Aperio Image scope version v11.2.0.780 (Aperio, Vista, CA, USA). Briefly, six to seven areas encompassing epithelial or stromal cells from each section were randomly selected. The integrated optical density (IOD) value for each selected area was calculated using the software.

### Statistical analysis

Statistical analyses to determine the significance of difference in the transcript levels between RL95-2 and HEC-1-A and also to determine that in the intensities of immunoreactive antigens on pre-receptive and receptive endometrial tissues were carried out using unpaired Student's t test. Analyses were carried out using GraphPad Prism (version 4.0, GraphPad Inc.; San Diego, CA). The level of significance was set at p<0.05.

## Results

### HGEx-ERdb

The database HGEx-ERdb currently contains 19,285 genes and is open for deposition of additional data by other investigators. The database can be queried to retrieve the expression status of the gene of interest in different stages of the menstrual cycle and various other conditions such as chemical or hormone treatment, gestation, contraception or pathologies. In addition, HGEx-ERdb provides information about the molecular features of genes or their cognate proteins (promoter sequence; amino acid sequence, location and molecular function of the encoded protein, interacting partners of the encoded protein).

### Receptivity Associated Genes (RAGs)

Analysis of 84 data sets (24 studies) available on the human endometrial gene expression revealed expression of 12,099 genes during the receptive phase (Figure 1). In contrast, 7289 genes appeared to be transcriptionally silent/repressed or less active in the receptive phase (as indicated by very low signal intensity in microarray hybridizations). These genes were scored for their expression status and also for their expression pattern (Tables 1, 2, 3) in the receptive phase. For 12,099 expressed genes in the receptive phase, the scores were in the range of 2–16. When scored for the expression pattern, 159 genes were upregulated in the receptive phase compared with the pre-receptive phase, with scores in the range of 2–18. Cumulative scoring led to the identification of 151 genes (Up-Ex genes) with score ranging from 4 to 30. Similarly, cumulative scoring of 7289 genes identified as “not-detected”, and 125 downregulated genes (scores 2–6) yielded 28 Down-Nd genes, which displayed downregulation in the receptive phase as compared to the pre-receptive phase. The cumulative scores for the Down-Nd RAGs ranged from 4 to 14 (Table 4).

**Table 1:** Up-Ex Receptivity Associated Genes (RAGs) and their Reliability Scores.

S.No.	Gene Symbol	Gene Name	Upregulation Score	Expression Score	Cumulative Score
1	SPP1	secreted phosphoprotein 1	18	12	30
2	GPX3	glutathione peroxidase 3 (plasma)	14	12	26
3	PAEP	progestagen-associated endometrial protein	12	12	24
4	IGFBP7	insulin-like growth factor binding protein 7	12	12	24
5	IL15	interleukin 15	12	12	24
6	CD55	CD55 molecule, decay accelerating factor for complement (Cromer blood group)	10	12	22
7	CLDN4	claudin 4	6	16	22
8	DPP4	dipeptidyl-peptidase 4	8	12	20
9	COMP	cartilage oligomeric matrix protein	6	12	18
10	LAMB3	laminin, beta 3	6	12	18
11	TIMP1	TIMP metallopeptidase inhibitor 1	4	14	18
12	DCN	decorin	4	14	18
13	LIF	leukemia inhibitory factor (cholinergic differentiation factor)	2	16	18
14	TCN1	transcobalamin I (vitamin B12 binding protein, R binder family)	4	12	16
15	C4BPA	complement component 4 binding protein, alpha	4	12	16
16	IL6ST	interleukin 6 signal transducer (gp130, oncostatin M receptor)	4	12	16
17	MAOA	monoamine oxidase A	4	12	16
18	MFAP5	microfibrillar associated protein 5	4	12	16
19	TSPAN8	tetraspanin 8	4	12	16
20	FAM148B	family with sequence similarity 148, member B	4	12	16
21	GADD45A	growth arrest and DNA-damage-inducible, alpha	4	12	16
22	S100P	S100 calcium binding protein P	4	12	16
23	IGFBP3	insulin-like growth factor binding protein 3	4	12	16
24	FXYD2	FXYD domain containing ion transport regulator 2	4	12	16
25	ABCC3	ATP-binding cassette, sub-family C (CFTR/MRP), member 3	4	12	16
26	TIMP2	TIMP metallopeptidase inhibitor 2	4	12	16
27	ANGPTL1	angiopoietin-like 1	4	12	16
28	WFDC2	WAP four-disulfide core domain 2	2	14	16
29	AIMP1	aminoacyl tRNA synthetase complex-interacting multifunctional protein 1	2	12	14
30	TMOD1	tropomodulin 1	2	12	14
31	IL1R1	interleukin 1 receptor, type I	2	12	14
32	SNX10	sorting nexin 10	2	12	14
33	UBE2L6	ubiquitin-conjugating enzyme E2L 6	2	12	14
34	CXCR4	chemokine (C-X-C motif) receptor 4	2	12	14
35	CYP3A5	cytochrome P450, family 3, subfamily A, polypeptide 5	2	12	14
36	HLA-A	major histocompatibility complex, class I, A	2	12	14
37	GJA1	gap junction protein, alpha 1, 43kDa	2	12	14
38	ANG	angiogenin, ribonuclease, RNase A family, 5	2	12	14
39	CD36	CD36 molecule (thrombospondin receptor)	2	12	14
40	SLC1A1	solute carrier family 1	2	12	14
41	DHRS3	dehydrogenase/reductase (SDR family) member 3	2	12	14
42	SFN	Stratifin	2	12	14
43	NID1	nidogen 1	2	12	14
44	TGM2	transglutaminase 2 (C polypeptide, protein-glutamine-gamma-glutamyltransferase)	2	12	14
45	ANXA4	annexin A4	2	12	14
46	C1orf106	chromosome 1 open reading frame 106	2	12	14
47	PTN	pleiotrophin	2	12	14
48	BCL2A1	BCL2-related protein A1	2	12	14
49	ANXA2	annexin A2 pseudogene 3; annexin A2; annexin A2 pseudogene 1	2	12	14
50	RARRES1	retinoic acid receptor responder (tazarotene induced) 3	2	12	14
51	G0S2	G0/G1switch 2	2	12	14
52	THBD	thrombomodulin	2	12	14
53	FBLN1	fibulin 1	2	12	14
54	CYP2C9	cytochrome P450, family 2, subfamily C, polypeptide 9	2	12	14
55	CTTN	cortactin	2	12	14
56	PAPSS2	3′-phosphoadenosine 5′-phosphosulfate synthase 2	2	12	14
57	B2M	beta-2-microglobulin	2	12	14
58	ADRA2A	adrenergic, alpha-2A-, receptor	2	12	14
59	MAP3K5	mitogen-activated protein kinase kinase kinase 5	2	12	14
60	C9orf71	chromosome 9 open reading frame 71	2	12	14
61	DKK1	dickkopf homolog 1 (Xenopus laevis)	2	12	14
62	ST6GAL1	ST6 beta-galactosamide alpha-2,6-sialyltranferase 1	2	12	14
63	GRAMD1C	GRAM domain containing 1C	2	12	14

**Table 2:** Up-Ex Receptivity Associated Genes (RAGs) and their Reliability Scores.

S.No.	Gene Symbol	Gene Name	Upregulation Score	Expression Score	Cumulative Score
64	C1S	complement component 1, s subcomponent	2	12	14
65	EDNRB	endothelin receptor type B	2	12	14
66	IGFBP5	insulin-like growth factor binding protein 5	2	12	14
67	GDF15	growth differentiation factor 15	2	12	14
68	CXCL13	chemokine (C-X-C motif) ligand 13	2	12	14
69	TIMP3	TIMP metallopeptidase inhibitor 3	2	12	14
70	CLU	clusterin	2	12	14
71	NP	ortholog of mouse neuropoietin (pseudogene in humans)	2	12	14
72	SLC44A4	solute carrier family 44, member 4	2	12	14
73	AQP3	aquaporin 3 (Gill blood group)	2	12	14
74	FGFR2	fibroblast growth factor receptor 2	2	12	14
75	SCGB2A2	secretoglobin, family 2A, member 2	2	12	14
76	THBS2	thrombospondin 2	2	12	14
77	PPAP2B	phosphatidic acid phosphatase type 2B	2	12	14
78	TNFAIP2	tumor necrosis factor, alpha-induced protein 2	2	12	14
79	HAL	histidine ammonia-lyase	2	12	14
80	APOD	apolipoprotein D	2	12	14
81	TAGLN	transgelin	2	12	14
82	MFGE8	milk fat globule-EGF factor 8 protein	2	12	14
83	NUPR1	nuclear protein, transcriptional regulator, 1	2	12	14
84	GPR110	G protein-coupled receptor 110	2	12	14
85	PDZK1IP1	PDZK1 interacting protein 1	2	12	14
86	NFIL3	nuclear factor, interleukin 3 regulated	2	12	14
87	CXCL12	chemokine (C-X-C motif) ligand 12 (stromal cell-derived factor 1)	2	12	14
88	CXCL14	chemokine (C-X-C motif) ligand 14	2	12	14
89	ELF3	E74-like factor 3 (ets domain transcription factor, epithelial-specific)	2	12	14
90	CATSPERB	cation channel, sperm-associated, beta	2	12	14
91	COL15A1	collagen, type XV, alpha 1	2	12	14
92	FAM148A	family with sequence similarity 148, member A	2	12	14
93	PTGER2	prostaglandin E receptor 2 (subtype EP2), 53kDa	2	12	14
94	LMOD1	leiomodin 1 (smooth muscle)	2	12	14
95	THBS1	thrombospondin 1	2	12	14
96	APOL1	apolipoprotein L, 1	2	12	14
97	HABP2	hyaluronan binding protein 2	2	12	14
98	AOX1	aldehyde oxidase 1	2	12	14
99	DNAJC6	DnaJ (Hsp40) homolog, subfamily C, member 6	2	12	14
100	PROM1	prominin 1	2	12	14
101	AGR2	anterior gradient homolog 2 (Xenopus laevis)	2	12	14
102	SOD2	superoxide dismutase 2, mitochondrial	2	12	14
103	SLC15A1	solute carrier family 15 (oligopeptide transporter), member 1	2	12	14
104	MT1H	metallothionein 1H	2	12	14
105	IMPA2	inositol(myo)-1(or 4)-monophosphatase 2	2	12	14
106	ACADSB	acyl-Coenzyme A dehydrogenase, short/branched chain	2	12	14
107	BTBD3	BTB (POZ) domain containing 3	2	12	14
108	KIAA1199	colon cancer secreted protein 1; protein KIAA1199	2	12	14
109	EFEMP1	EGF-containing fibulin-like extracellular matrix protein 1	2	12	14
110	VPS13D	vacuolar protein sorting 13 homolog D (S. cerevisiae)	2	12	14
111	OTUB2	OTU domain, ubiquitin aldehyde binding 2	2	12	14
112	TSPO	translocator protein (18kDa)	2	12	14
113	ARID5B	AT rich interactive domain 5B (MRF1-like)	2	12	14
114	MT1E	metallothionein 1L (gene/pseudogene); metallothionein 1E;	2	12	14
115	F3	coagulation factor III (thromboplastin, tissue factor)	2	12	14
116	C1R	complement component 1, r subcomponent	2	12	14
117	PAX8	paired box 8	2	12	14
118	ANKRD55	ankyrin repeat domain 55	2	12	14
119	FOXO1	forkhead box O1	2	12	14
120	VCAN	versican	2	12	14
121	TRPM8	transient receptor potential cation channel, subfamily M, member 8	2	12	14
122	MT1G	metallothionein 1G	2	12	14
123	SLC22A5	solute carrier family 22 (organic cation/carnitine transporter), member 5	2	12	14
124	AHNAK	AHNAK nucleoprotein	2	12	14
125	KIAA0040	uncharacterized protein KIAA0040	2	12	14
126	SLPI	secretory leukocyte peptidase inhibitor	2	12	14
127	RARRES3	retinoic acid receptor responder (tazarotene induced) 3	2	12	14
128	DUSP6	dual specificity phosphatase 6	2	12	14
129	ACTA2	actin, alpha 2, smooth muscle, aorta	2	12	14
130	PLS1	plastin 1 (I isoform)	2	12	14

**Table 3:** Up-Ex Receptivity Associated Genes (RAGs) and their Reliability Scores.

S.No.	Gene Symbol	Gene Name	Upregulation Score	Expression Score	Cumulative Score
131	TTC39A	tetratricopeptide repeat domain 39A	2	12	14
132	GAST	gastrin	2	12	14
133	IGFBP1	insulin-like growth factor binding protein 1	4	8	12
134	DARC	Duffy blood group, chemokine receptor	4	8	12
135	LCP1	lymphocyte cytosolic protein 1 (L-plastin)	2	10	12
136	MMP11	matrix metallopeptidase 11 (stromelysin 3)	2	10	12
137	TRPC6	transient receptor potential cation channel, subfamily C, member 6	2	10	12
138	PSD	pleckstrin and Sec7 domain containing	2	10	12
139	PPARGC1A	peroxisome proliferator-activated receptor gamma, coactivator 1 alpha	2	8	10
140	ABLIM3	actin binding LIM protein family, member 3	2	8	10
141	CDH13	cadherin 13, H-cadherin (heart)	2	8	10
142	FAP	fibroblast activation protein, alpha	2	8	10
143	ATP2C2	ATPase, Ca++ transporting, type 2C, member 2	2	8	10
144	MUC16	mucin 16, cell surface associated	2	8	10
145	LCN2	lipocalin 2	2	6	8
146	CDA	cytidine deaminase	2	6	8
147	ABP1	amiloride binding protein 1 (amine oxidase (copper-containing))	2	4	6
148	FGB	fibrinogen beta chain	2	4	6
149	MYH11	myosin, heavy chain 11, smooth muscle	2	4	6
150	CNN1	calponin 1, basic, smooth muscle	2	4	6
151	RBP4	retinol binding protein 4	2	2	4

**Table 4:** Down-Nd Receptivity Associated Genes (RAGs) and their Reliability Scores.

S.No.	Gene Symbol	Gene name	Downregulation score	Not-detected score	Cumulative score
1	E2F2	E2F transcription factor 2	2	12	14
2	CDC45L	cell division cycle 45 homolog L	2	12	14
3	BMP7	bone morphogenetic protein 7	2	12	14
4	KCNG1	potassium voltage gated channel, subfamily G, member 1	2	12	14
5	S100Z	S100 calcium binding protein Z	2	12	14
6	EFNA2	ephrin A2	2	12	14
7	S100A2	S100 calcium binding protein A2	2	12	14
8	S100G	S100 calcium binding protein G	2	12	14
9	PLA1A	phospholipase A1 member A	2	12	14
10	S100A5	S100 calcium binding protein A5	2	12	14
11	S100B	S100 calcium binding protein B	2	12	14
12	EPHB3	EPH receptor B3	4	8	12
13	TRH	thyrotropin releasing hormone	2	10	12
14	FOXM1	forkhead box M1	2	10	12
15	S100A7A	S100 calcium binding protein A7A	2	10	12
16	S100A7	S100 calcium binding protein A7	2	10	12
17	GJB6	gap junction protein, beta 6, 30kDa	2	10	12
18	TACC3	transforming, acidic coiled coil containing protein 3	2	8	10
19	CDC20	cell division cycle 20 homolog (S. cerevisiae)	4	4	8
20	PTTG1	pituitary tumor transforming 1; pituitary tumor transforming 2	4	4	8
21	KIF20A	kinesin family member 20A	2	4	6
22	PAQR4	progestin and adipoQ receptor family member IV	2	4	6
23	CALB2	calbindin 2	2	4	6
24	CENPE	centromere protein E, 312kDa	2	4	6
25	GALNT12	UDP N acetyl alpha D galactosamine:polypeptide N acetylgalactosaminyltransferase 12 (GalNAc T12)	2	4	6
26	CENPA	centromere protein A	2	2	4
27	SLC26A4	solute carrier family 26, member 4	2	2	4
28	GREM2	gremlin 2, cysteine knot superfamily, homolog (Xenopus laevis)	2	2	4

### Expression of RAGs in women with IVF failure

Analysis of the available two data sets of endometrial gene expression in ten women, who had previously experienced IVF failure, indicated that 12,799 genes were transcribed and 6486 appeared to be not detected. In these women, 13 genes were found to have lesser expression during the receptive phase, compared to healthy women ( Table S1).

### Gene Ontology (GO) Analysis of RAGs

**Figure 2:**
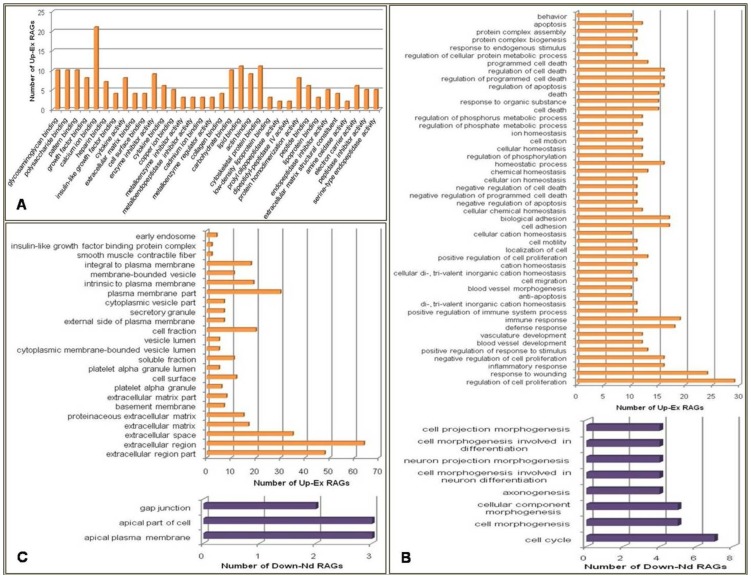
Gene Ontology (GO) analysis to categorize the Receptivity Associated Genes (RAGs) according to A) molecular functions B) biological processes C) cellular components. Only significant (p<0.05) annotations are shown. UP-EX genes enriched various biological processes such as regulation of cell proliferation, response to wounding, immune response, cell adhesion and cellular, chemical homeostasis, regulation of cell death and blood vessel development. These included 160 biological processes. Only those biological processes with gene count of at least 10 are shown in panel B.

RAGs were classified with Gene Ontology (GO) analysis according to molecular function, biological process and cellular component using DAVID tool. The ‘molecular functions’ found associated with the Up-Ex RAGs included calcium ion binding, glycosaminoglycan binding and cytoskeletal protein binding (Figure 2A). The major ‘biological processes’ mediated by Up-Ex RAGs were regulation of cell proliferation, response to wounding, immune response, cell adhesion and cellular and chemical homeostasis. Down-Nd RAGs were also found to be associated with calcium ion binding. The major ‘biological processes’ of these genes were cell cycle, cell morphogenesis and motility (Figure 2B). The majority of Up-Ex RAGs proteins was found to encode either extracellular or plasma membrane proteins (Figure 2C). Only those GO annotations which had a significant p-value <0.05 have been depicted in Figure 2.

Up-Ex RAGs could be functionally clustered into 33 groups and Down-Nd RAGs into 5 clusters (Table S2, S3). Major functional clusters for Up-Ex RAGs were glycosaminoglycan binding, cell migration, inorganic cation hemostasis, regulation of phosphorylation, regulation of apoptosis. Down-Nd RAGs were found in the clusters annotated as calcium binding region, domain EF hand, and mitosis.

### Regulation of the RAGs

GeneMANIA analysis demonstrated co-expression (89.99%) and co-localization (6.69%) as major relationships amongst Up-Ex RAGs Figure S2A). Down-Nd RAGs were also related to each other by co-expression (94.13%) as shown in Figure S2B. This suggested the possibility of co-regulation of RAGs by common transcription factors (TFs). To explore this, *in silico* analysis was carried out using GATHER [21] to identify the transcription factors which are probably shared by RAGs.

**Figure 3:**
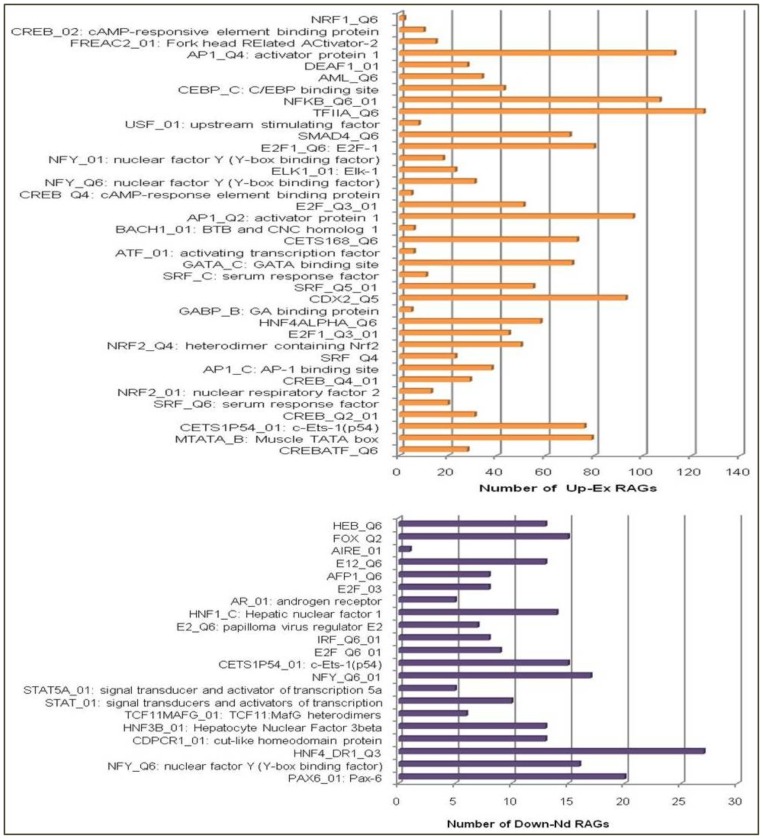
*In silico* analysis by GATHER to identify the transcription factor binding sites (TFBS) in the regulatory regions of receptivity associated genes (RAGs). (p<0.05).

The majority of Up-Ex RAGs had TFII, AP1, NFkB, CDX2 and CEBP binding sites, thereby suggesting the possibility of activation of these TFs during the receptive phase. TFII transcription factor binding site was found in the promoters of 125 of Up-Ex RAGs, while AP1, NFκB, CDX2 and CEBP in the promoters of 113, 107, 93 and 43 of Up-Ex RAGs respectively. HNF4 transcription factor binding site was present in 27, PAX6 in 20, NFY or the nuclear factor Y binding site was found in 17 of 28 Down-Nd RAGs (Figure 3). Interestingly, the genes coding for some of these transcription factors were also among the genes expressed in the receptive phase, such as NFkB2, NFkB1, AP1G1, AP1M1, CEBPG and CEBPD. This observation strengthens the possibility of these transcription factors activating the transcription of RAGs in the receptive phase.

### Experimental Validation of Select RAGs at the Transcript and Protein levels

As acquisition of the adhesiveness is a primary feature of the receptive endometrium, we focussed on validating the expression of those RAGs which encode cell adhesion proteins. Among the Up-Ex RAGs, THBS1, COMP, CD36, MUC16, SPP1, and DPP4 were chosen because of their established role in cell adhesion and also because of their high reliability scores. Further the majority of these genes (except SPP1 and MUC16) have not been investigated previously for their association with endometrial receptivity. Lower levels of COMP and MUC16 in women with IVF failure (as per HGEx-ERdb) also prompted us to select these two RAGs.

**Figure 4:**
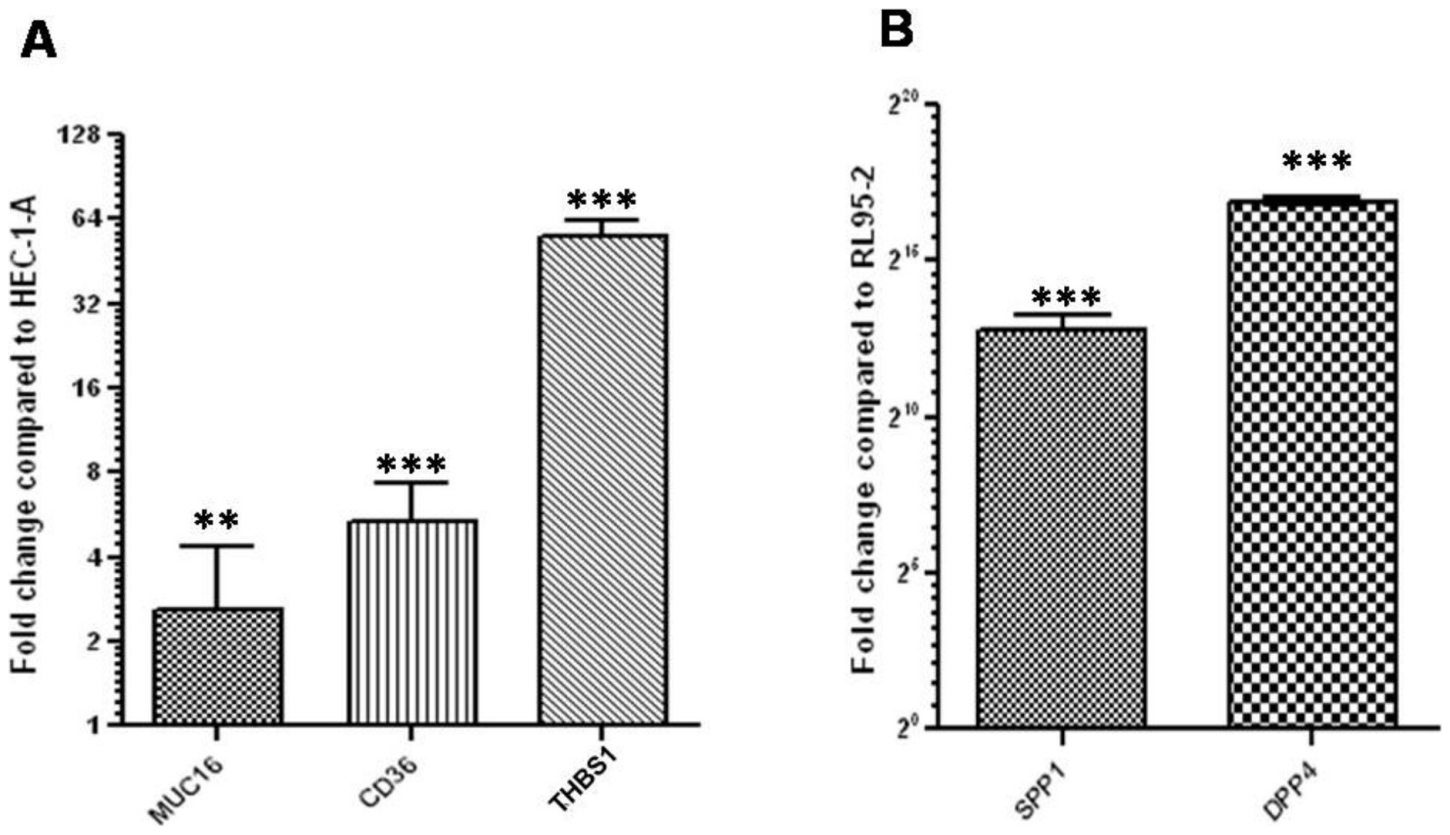
Validation of select RAGs by q-RTPCR in RL95-2 (a more adhesive cell line) and HEC-1-A (a less adhesive cell line). Relative levels of the transcripts for MUC16, CD36 and THBS1 in RL95-2 as compared to HEC-1-A, are shown in panel A. Panel B demonstrates relative levels of the transcripts for SPP1 and DPP4 in HEC-1-A as compared to RL95-2. ** p <0.0005, *** p <0.0002, p value indicates the significance of the difference.

RL95-2, a more adhesive cell line, had significantly higher (p<0.05) levels of THBS1, CD36 and MUC16 as compared to HEC-1-A, a less adhesive cell line (Figure 4A). However, the relative levels of SPP1 and DPP4 were significantly (p<0.05) lower in RL95-2 as compared to HEC-1-A (Figure 4B).

**Figure 5:**
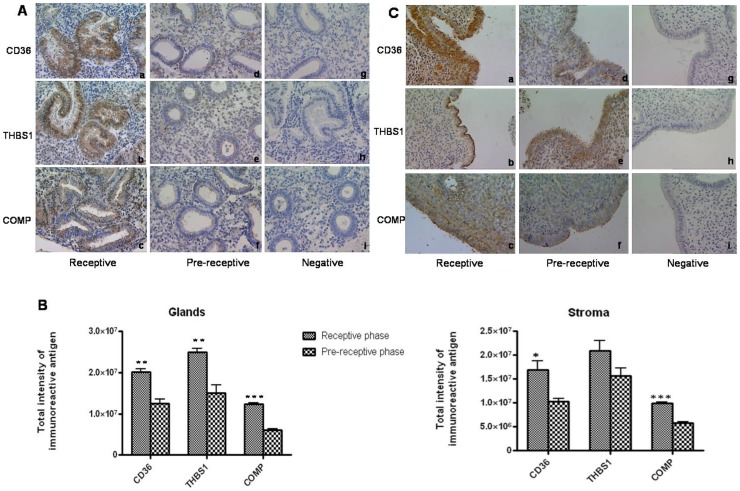
Immunolocalization of the proteins encoded by select RAGs in receptive and pre-receptive phase human endometrial tissues. A) Immunohistochemical localization of CD36, THBS1 and COMP in receptive phase (a,b,c) and pre-receptive phase (d,e,f) endometrial tissues. Panels g and i represent the sections stained with rabbit IgG and h shows the section stained with mouse IgG. B) Semiquantitative analysis to compare the intensities of immunoreactive CD36, THBS1 and COMP in epithelial and stromal compartments of receptive and pre-receptive phase human endometrial samples. *p<0.05, ** p <0.005, *** p<0.002 C) Immunohistochemical localization of CD36,THBS1 and COMP in the luminal epithelium of human endometrial tissues collected in receptive phase (a,b,c) and pre-receptive phase (d,e,f) of menstrual cycle. Panels g and i represent the sections stained with rabbit IgG and panel h is stained with mouse IgG. Magnification at 40X.

Immunohistochemical localization of THBS1,CD36 and COMP proteins demonstrated immunopositivity in the cytoplasmic compartment of the glandular epithelium and stroma of human endometrium (Figure 5A). However, intensities of immunolocalized proteins were remarkably higher in the epithelial compartment as compared to stromal compartment. Further intensities of immunoreactive proteins in endometrium were significantly higher (p<0.05) in the receptive phase as compared to that in pre-receptive phase (Figure 5B). This reiterated the validity of their placement in the list of RAGs. Luminal epithelia of endometrial tissues also demonstrated the presence of immunoreactive CD36, THBS1 and COMP (Figure 5C). Their intensities appeared to be higher in the receptive phase endometrium compared to pre-receptive endometrium. Presence of these proteins in the luminal epithelial compartment hinted at the possibility of their role in embryo adhesion.

**Figure 6:**
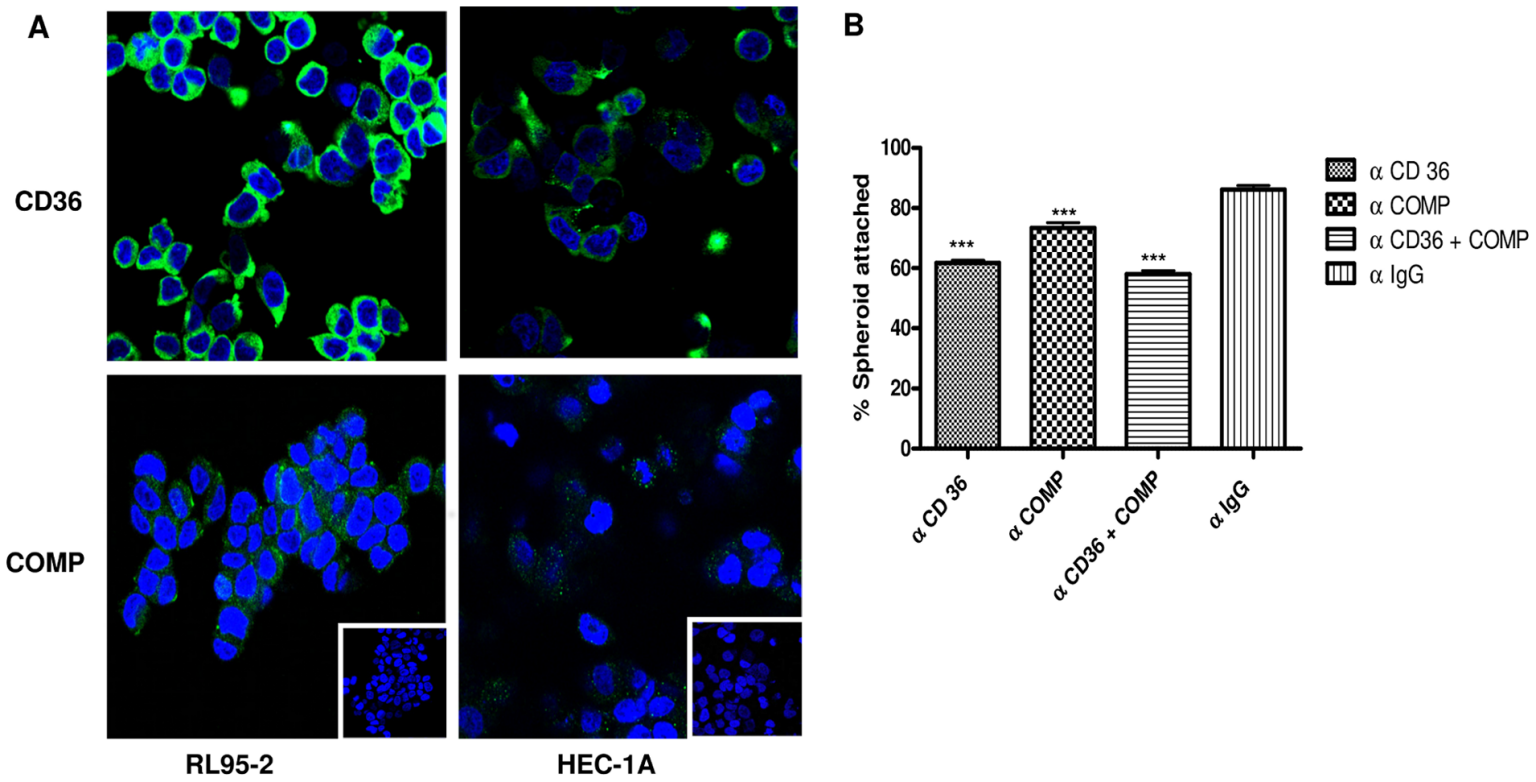
Immunofluorescent localization of CD36 and COMP proteins and their functional significance as assessed by in vitro spheroid attachment assays. Panel A shows cytoplasmic localization of CD36 and COMP in RL95-2 and HEC-1A. Respective negatives controls stained with rabbit IgGs are shown in the insets. Magnification: 63X. Panel B displays percentage spheroids attached to RL95-2 cells, pretreated with antibodies against CD36 or COMP or both. Percent spheroids attached to RL95-2 cells pretreated with same concentration of rabbit IgG are also shown (p<0.0002).

Confocal microscopy analysis revealed presence of CD36 and COMP on the cell surface of RL95-2 and HEC-1-A (Figure S3). Further immunofluorescence studies demonstrated higher intensities of immunoreactive CD36 and COMP in RL95-2 as compared to HEC-1-A (Figure 6A). Preincubation of RL95-2 cells with antibodies against CD36,COMP and CD36 combined with COMP led to a reduction in the percentage of spheroids attached (19.5%,12.83% and 28.16% respectively) to RL95-2 cells compared to those treated with same concentration of rabbit IgGs (Figure 6B). These observations were implicative of the possibility that these two molecules, especially CD36 play an important role in embryo-endometrial adhesion.

## Discussion

Embryo implantation is one of the most crucial steps that dictate the outcome of reproduction and hence has attracted the attention of several researchers engaged in pregnancy research. It is well established that embryo implantation is initiated only when the endometrium of uterus is hormone primed and appropriately transformed at structural and functional levels [27]. Endometrial transformation towards the receptivity is mediated by a large number of gene/gene products. Several investigations [4]–[11] have led to the identification of genes which are differentially expressed during the receptive period in menstrual cycle. Realizing the relevance of assimilating this information on a single platform and identifying those genes that display the similar status or pattern of expression in different datasets, the study was undertaken to create HGEx-ERdb.

HGEx-ERdb provides information about the expression of 19,285 genes in human endometrium. For the creation of this database, 312 data sets were retrieved from online resources such as GEO and 51 peer reviewed publications. HGEx-ERdb is a catalogue of all the genes, reported till date for their expression or repression in human endometrium, during various phases of the natural menstrual cycle or in other conditions including stimulated cycles.

HGEx-ERdb is the first database that stores endometrial gene expression data, particularly in the receptive phase, and allows context-specific queries. The database can be used to retrieve the following information/data:

- Expression status (in isolation or in comparison) of the gene of interest in endometrium.
- List of all the genes reported to be expressed in human endometrium in different phases of the menstrual cycle.
- Alterations in endometrial gene profile (specific/global) in response to hormone, chemical, COS cycle, IVF treatment or other disorders.
- Cellular localization, molecular function and role of the select gene in biological processes.
- Protein, transcript, promoter and protein- protein interactions of the selected gene.

Reliability score forms a semi quantitative method of deriving a consensus across different datasets irrespective of the technology, platform and availability of raw and processed data [12]. This score from HGEx-ERdb provides a means to select genes of higher significance for the conditions of interest. The links for functional analysis can also be useful in short-listing relevant genes.

Querying the HGEx-ERdb for endometrial gene signatures yielded 12,099 genes which are expressed and 7289 genes appear as not detected in the receptive phase endometrium. Out of these, 151 genes (Up-Ex) displayed the similar pattern of expression (upregulation) in the receptive phase as compared to pre-receptive phase across different datasets. Further, 28 genes (Down-Nd) were found to be downregulated in the receptive phase, when compared to pre-receptive phase.

The functional annotation clustering pointed that 62.25% of the Up-Ex RAGs encode the extracellular and plasma membrane proteins. This reinforces the relevance of optimal expression of cell surface and extracellular matrix proteins in endowing the endometrium with receptivity, as these proteins may be of prime importance in embryo adhesion and attendant signal transduction pathways.

Up-Ex RAGs are known to regulate cytokine-cytokine interaction pathway, complement and coagulation cascades, ECM-receptor interaction and inhibition of matrix-metalloproteinase pathway. Activation of these pathways during the receptive phase may equip the endometrium for structural and functional modifications, required for embryo attachment and growth.

In the list of Down-Nd RAGs, predominant were the genes associated with cell cycle regulation. This was implicative of decreased mitotic activity in the endometrium during the receptive phase. It is well established that endometrial receptivity is marked by cellular differentiation of the functional layer of endometrium. This probably explains downregulation in the expression of genes associated with cell cycle regulation during the receptive phase. An interesting observation was the downregulation of many members of the S100 protein family, during the receptive phase. S100 proteins, small acidic proteins of 10–12 kDa with calcium binding EF hand (helixE-loop-helixF) motifs, regulate variety of cellular functions such as cell growth and differentiation, cell cycle progression, protein phosphorylation and secretion etc. [28]. Their lesser expression during the receptive phase may regulate proliferative activity of endometrial cells.

Analysis of transcription factor binding sites (TFBS) in the regulatory regions demonstrated overrepresentation of TFII, AP1, NFkB, CDX2, CEBP binding sites in Up-Ex RAGs and that of HNF4, NFY, PAX6 in Down-Nd RAGs. Tapia et al [14] have also demonstrated the predominance of AP1, HNF4, NFY binding sites in the genes displaying differential expression during the receptive phase. However, their analysis was based on a limited number of datasets. It will be interesting to investigate whether predicted TFBSs are functional during the receptive phase and if yes, which posttranscriptional or posttranslational mechanisms are involved in the activation of transcription factors, presumably binding to TFBS of RAGs.

Endometrium acquires adhesiveness to an embryo only during the receptive phase and hence it was not surprising to note that most of the RAGs encode extracellular and plasma membrane proteins. This was implicative of the critical role played by genes which encode adhesive proteins. THBS1, CD36, COMP, SPPI, DPP4 and MUC16, all known for their role in cell adhesion, were chosen for the experimental validation using two human endometrial epithelial cell lines RL95-2 and HEC-1-A. Although these immortalized cell lines do not truly represent pre-receptive and receptive phase primary endometrial tissues, these were selected as experimental cell models for the validation of transcription pattern of RAGs, for two reasons. First, these cell lines are known for their differential adhesiveness to embryonic cells and second, human endometrial RNA samples were not available. Further THBS1, CD36 and COMP were selected for validation in tissues (stored paraffin sections of human endometrium) by immunolocalization, as these have not been investigated previously for their expression at protein level during the receptive phase.

Interestingly, 3 members of the thrombospondin family i.e. THBS1, THBS2 and THBS5 (COMP) appeared as Up-Ex RAGs in the present study. Thrombospondins (TSPs) are modular proteins which contain globular domains at their amino and carboxyl terminals, EGF like type 2 and calcium binding type 3 repeat domains [29]. THBS1 is a large trimeric extracellular matrix protein secreted by various cell types and has been shown to interact with more than 30 cell surface molecules and matrix proteins. THBS1 mediates adhesion and migration of cells, cellular growth, platelet aggregation and angiogenesis [30], [31]. Kawano et al [32] demonstrated the expression of THBS1 in endometrial stromal cells. However, no data are available on the expression pattern of TSP proteins during the receptive phase in human endometrium. Present study, though carried out in a limited number of human samples, demonstrated higher expression of endometrial THBS1 and THBS5 (COMP) in the receptive phase, compared to pre-receptive phase. Further aberrant expression of endometrial COMP in women who undergo IVF failure, provides a circumstantial evidence of the role of TSPs in embryo implantation.

Interacting partners or receptors of THBS1 include structural proteins like collagen, fibronectin, cell surface receptors- integrins, syndecans, enzymes like elastase and cytokines such as TGFβ1, in addition to CD36 or fatty acid translocase (FAT). THBS1 binds to surface receptors such as CD36 and initiates signalling to inhibit angiogenesis and cell migration [31]. Interestingly, CD36 was also found in the list of Up-Ex RAGs. Our immunohistochemical studies on the human endometrium also validated higher expression of CD36 in the receptive phase, compared to the pre-receptive phase. Also CD36 expression at transcript as well as protein levels was higher in RL95-2, a more adhesive cell line; compared to HEC-1-A, a less adhesive cell line. Thus endometrial receptivity appears to be accompanied by upregulation in the expression of anti-angiogenic genes (CD36 and THBSs) and also downregulation in the expression of cell cycle associated genes. This occurs probably to facilitate the regulation of angiogenesis and proliferation in endometrial cells during the receptive phase. In addition, CD36 may be of some relevance in embryo-endometrial adhesion, as indicated by in vitro spheroid attachment assays. Treatment of RL95-2 cells with antibodies against CD36 led to a reduction in the percentage of spheroids attached. Localization in the luminal epithelium also strengthens the possibility that endometrial CD36 plays a role in embryo adhesion.

Osteopontin 1 (SPP1) and Dipeptidyl Peptidase (DPP4) scored high, as adjudged by their reliability score, for consensus on their higher expression during the receptive phase. Unexpectedly their transcript levels were found lower in more adhesive RL95-2 cell line as compared to less adhesive HEC-1-A cell line, both of epithelial origin. It may be hypothesized that the endometrial expression of SPP1 and DPP4 is increased during the receptive phase, in response to signalling from the stromal compartment of endometrial tissue. On the other hand, it may also be inferred that SPP1 and DPP4 are not the absolute determinants for the embryo adhesiveness. Indeed, no significant difference has been found in the expression of SPP1 and its receptor between fertile and infertile women [33]. SPP1 was found to be associated with endometrial maturation; however its immunohistochemical assessment did not offer great benefit as compared to the histological dating [34].

It was also observed that 13 out of 151 Up-Ex RAGs are downregulated in the endometrium of the women who experienced IVF failure during the receptive phase. This suggested that optimal expression of these 13 genes (or some of these) in the endometrium may be crucial for embryo attachment. Indeed there exist several reports demonstrating the seminal role of some of these genes (such as LIF) in the initiation of pregnancy [35]. However other genes in this list have not been investigated to the same extent in context of their role in endometrial receptivity or embryo attachment. These genes should be explored in detail for their functional relevance in endometrial receptivity and implantation. We could not detect COMP transcripts in RL95-2 and HEC-1-A cell lines, despite using high amounts of cDNAs. However, endometrial COMP protein was found significantly higher in the receptive phase as compared to the pre-receptive phase in healthy women. It is likely that higher expression of endometrial COMP facilitates embryo adhesion and its aberrant expression during the receptive phase leads to implantation failure, as observed in women who undergo IVF failure. Although our in vitro experiments demonstrated only 12.83% decrease in the spheroid attachment to the endometrial epithelial cells pretreated with antibodies against COMP this cannot be disregarded, considering that embryo-endometrial adhesion may involve multiple cell adhesion proteins and deficiency in the expression of any of these proteins may adversely affect implantation.

MUC16 transcript levels were also found higher in RL95-2 as compared to HEC-1-A cell line. MUC16 is a membrane associated mucin with heavily glycosylated ectodomain and short cytoplasmic tail [36]. It is believed that its ectodomain contributes to the formation of a non-adhesive barrier. Indeed it has been shown that MUC16 protein is lost from the luminal epithelium of the endometrium during the receptive phase, to facilitate embryo adhesion [37]. On the other hand, evidences exist to suggest that the glycosylation pattern of MUC-1 in the receptive phase differs from that in the proliferative phase [38]–[40]. It is likely that similar post-translational modifications in the glycosylation pattern of MUC16 regulate adhesiveness of the endometrium to embryo. HGEx-ERdb revealed an increase in the endometrial MUC16 transcript levels in the receptive phase. To explain the need for increased transcription of *MUC16* gene which encodes an anti-adhesive protein, it may be hypothesized that either its anti-adhesive property is modulated during the receptive phase or it performs functions other than anti-adhesion. Different domains of MUC16 protein are known to serve different functions. The cytoplasmic tail of MUC16 may mediate certain signalling functions required for embryo implantation. Endometrial MUC16 transcripts were found to be lower in the women who undergo IVF failure (Table S1). Signalling deficits due to poor expression of endometrial MUC16 could contribute to IVF failure.

In brief, the study has generated a valuable research resource, the Human Gene Expression Endometrial Receptivity database (HGEx-ERdb). The study also identifies a set of receptivity associated genes. Some of the RAGs may have subjugate role and their expression may be critical for endowing the endometrium with the receptivity (causal relationship), while others may have redundant role. Investigations using human endometrial epithelial cell lines as experimental models and endometrial tissues proved association of some of these RAGs with receptivity. *In silico* functional analysis of the RAGs derived from the database showed well-defined relationships, co-expressions and common transcription factors binding sites. All these were indicative of the strong potential of the approach employed in this study. The compilation of gene-expression data sets, and a computational scoring method have helped in identifying 179 receptivity associated genes and also a subset of 13 genes which are suboptimally expressed in the endometrium of women who underwent IVF failure. Further investigations focused on delineation of the functions of RAGs in the endometrial context will provide significant insights into the mechanisms underlying human endometrial receptivity and early pregnancy losses in humans.

## Supporting Information

Figure S1 **Percentage JAr spheroids attached to RL95-2 and HEC-1-A cells.** Please note differential adhesiveness of RL95-2 and HEC-1-A to JAar spheroids. (*** p<0.0001).
(TIF)

Figure S2 **Relationship among Up-Ex (A) and Down-Nd (B) RAGs as predicted by GeneMANIA.**
(TIF)

Figure S3 **Confocal microscopic analysis showing Z optical sections of immunoreactive CD36 (A) and COMP (B) in RL95-2 and HEC-1-A Magnification: 63x; DIC images shown as insets.**
(TIF)

Table S1 **Genes displaying suboptimal endometrial expression during the receptive phase in women who undergo IVF failure.**
(DOCX)

Table S2 **Genes displaying suboptimal endometrial expression during the receptive phase in women who undergo IVF failure.**
(DOCX)

Table S3 **Functional annotation of Down-Rep Down-Nd RAGs using DAVID software.**
(DOCX)
