# Supplementary figures and images for: Endometrial Receptivity: A Revisit to Functional Genomics Studies on Human Endometrium and Creation of HGEx-ERdb
Source: PLoS One. 2013 Mar 26;8(3):e58419. doi: 10.1371/journal.pone.0058419 (PMC3608645; doi:10.1371/journal.pone.0058419)

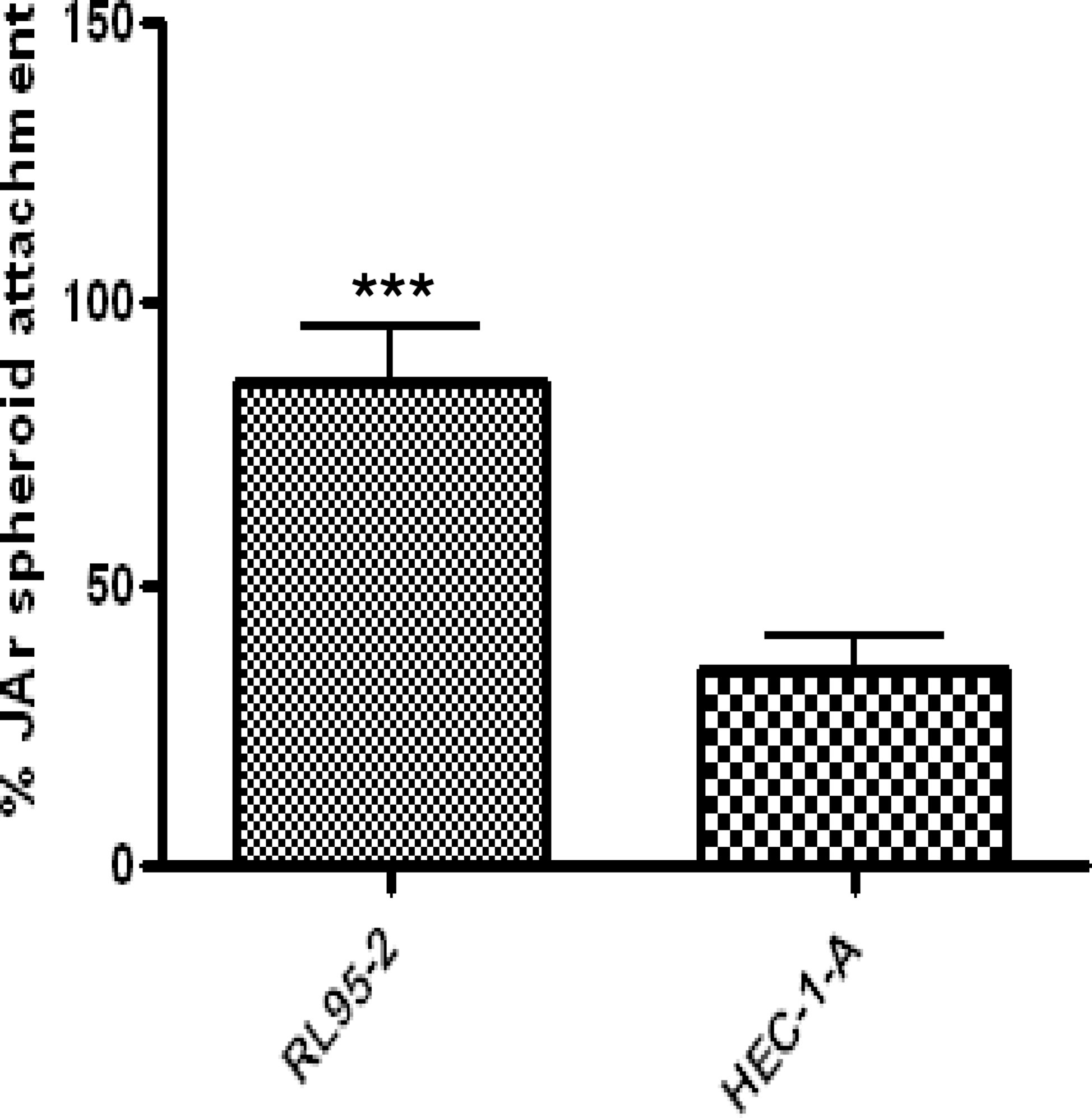

Supplement: Figure S1 — Percentage JAr spheroids attached to RL95-2 and HEC-1-A cells. Please note differential adhesiveness of RL95-2 and HEC-1-A to JAar spheroids. (*** p<0.0001). (TIF) [file pone.0058419.s001.tif]

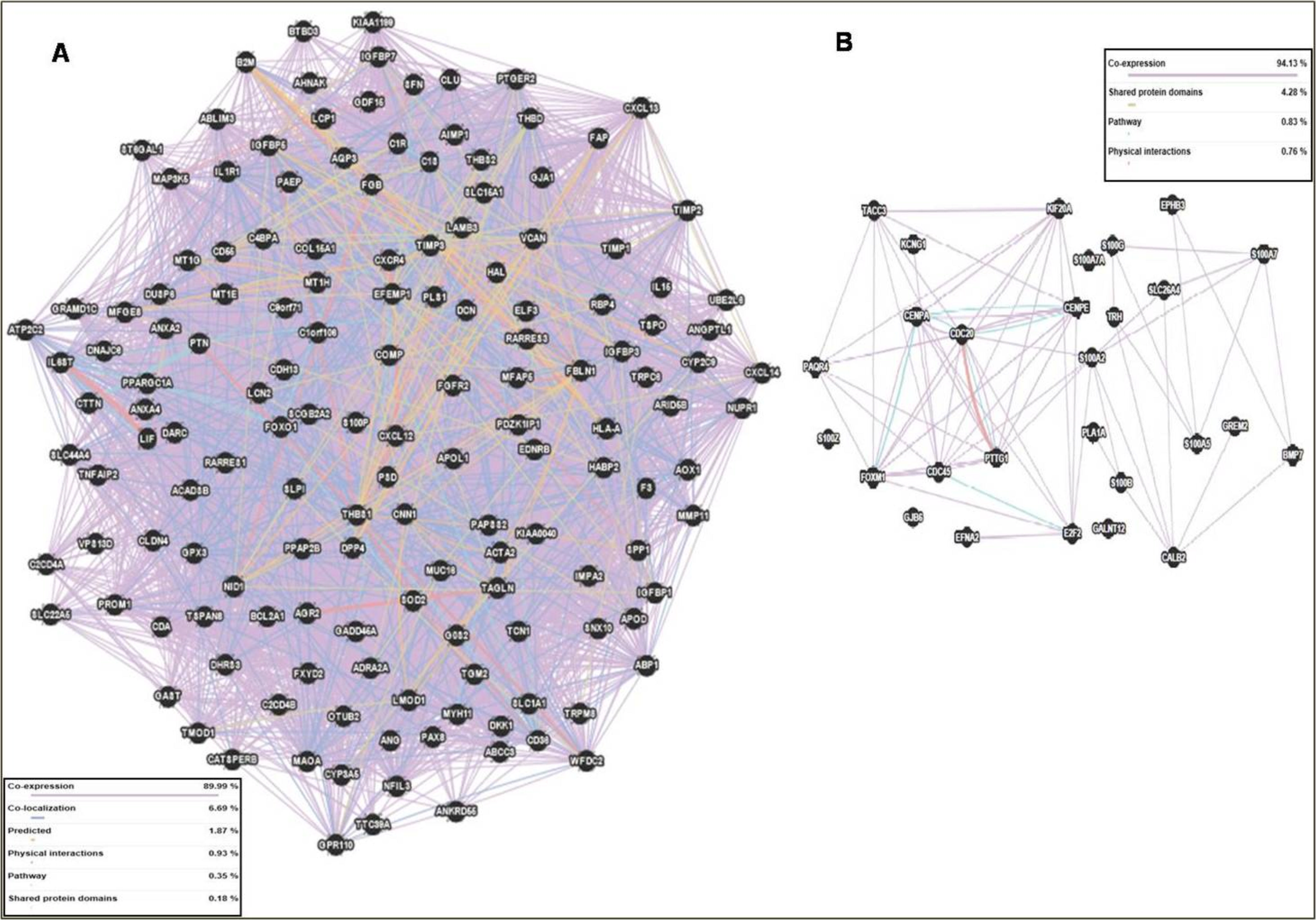

Supplement: Figure S2 — Relationship among Up-Ex (A) and Down-Nd (B) RAGs as predicted by GeneMANIA. (TIF) [file pone.0058419.s002.tif]

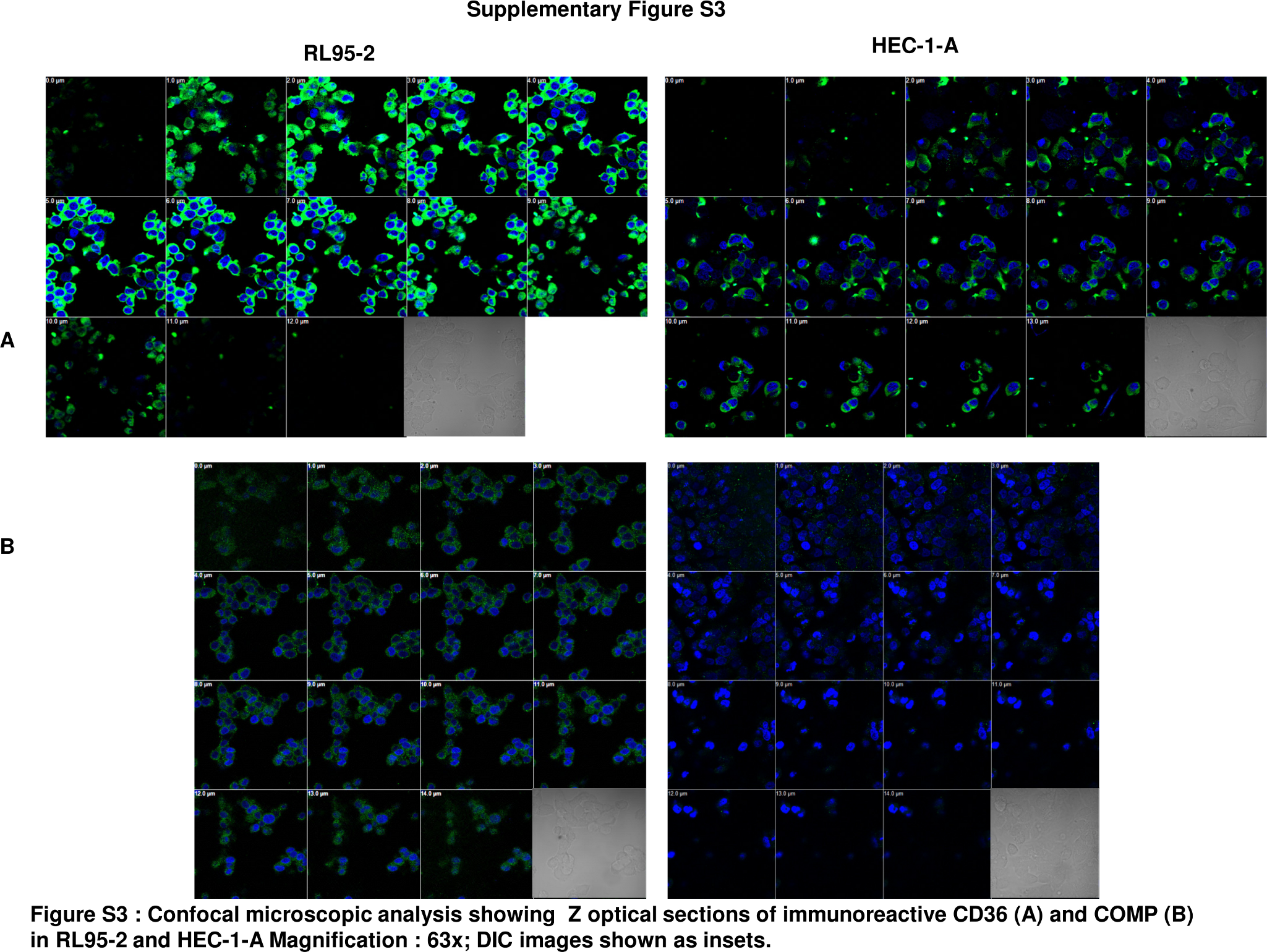

Supplement: Figure S3 — Confocal microscopic analysis showing Z optical sections of immunoreactive CD36 (A) and COMP (B) in RL95-2 and HEC-1-A Magnification: 63x; DIC images shown as insets. (TIF) [file pone.0058419.s003.tif]
