# Supplementary material for: Endometrial Receptivity: A Revisit to Functional Genomics Studies on Human Endometrium and Creation of HGEx-ERdb
Source: PLoS One. 2013 Mar 26;8(3):e58419. doi: 10.1371/journal.pone.0058419 (PMC3608645; doi:10.1371/journal.pone.0058419)
Supplement: Table S1 — Genes displaying suboptimal endometrial expression during the receptive phase in women who undergo IVF failure. (DOCX) [file pone.0058419.s004.docx]

**Table S1: Genes displaying suboptimal endometrial expression during the receptive phase in women who undergo IVF failure**

| **S.No.** | **Gene Symbol** | **Gene Name** | **Cumulative Score for higher expression in the receptive phase in healthy women** |
| --- | --- | --- | --- |
| 1 | COMP | CD55 molecule, decay accelerating factor for complement (Cromer blood group) | 18 |
| 2 | GJA1 | gap junction protein, alpha 1, 43kDa | 14 |
| 3 | GPR110 | G protein-coupled receptor 110 | 14 |
| 4 | TSPO | translocator protein (18kDa) | 14 |
| 5 | DARC | Duffy blood group, chemokine receptor | 12 |
| 6 | IGFBP1 | insulin-like growth factor binding protein 1 | 12 |
| 7 | ABLIM3 | actin binding LIM protein family, member 3 | 10 |
| 8 | MUC16 | mucin 16, cell surface associated | 10 |
| 9 | LCN2 | lipocalin 2 | 8 |
| 10 | ABP1 | amiloride binding protein 1 (amine oxidase (copper-containing)) | 6 |
| 11 | CNN1 | calponin 1, basic, smooth muscle | 6 |
| 12 | FGB | fibrinogen beta chain | 6 |
| 13 | MYH11 | myosin, heavy chain 11, smooth muscle | 6 |
