# Supplementary material for: Endometrial Receptivity: A Revisit to Functional Genomics Studies on Human Endometrium and Creation of HGEx-ERdb
Source: PLoS One. 2013 Mar 26;8(3):e58419. doi: 10.1371/journal.pone.0058419 (PMC3608645; doi:10.1371/journal.pone.0058419)
Supplement: Table S2 — Genes displaying suboptimal endometrial expression during the receptive phase in women who undergo IVF failure. (DOCX) [file pone.0058419.s005.docx]

**Table S2: Functional annotation of Up-Ex RAGs using DAVID software (p<0.05)**

| **Annotation Cluster Number** | **Enrichment Score** | **Category** | **Term** | **Count** | **PValue** | **Genes** |
| --- | --- | --- | --- | --- | --- | --- |
| 1 | 4.707866 | INTERPRO | IPR001881:EGF-like calcium-binding | 8 | 1.79E-05 | FBLN1, THBD, COMP, EFEMP1, C1R, VCAN, NID1, C1S |
| 2 | 4.497908 | GOTERM_MF_FAT | GO:0005539~glycosaminoglycan binding | 10 | 3.78E-06 | FGFR2, ABP1, ANG, COMP, PTN, VCAN, DCN, THBS1, THBS2, HABP2 |
| 3 | 3.5659 | UP_SEQ_FEATURE | domain:Thyroglobulin type-1 | 4 | 1.20E-04 | NID1, IGFBP1, IGFBP3, IGFBP5 |
| 4 | 3.379343 | GOTERM_BP_FAT | GO:0006874~cellular calcium ion homeostasis | 9 | 2.94E-04 | EDNRB, CD55, TRPM8, TRPC6, CXCR4, CXCL13, IL6ST, TGM2, CXCL12 |
| 5 | 3.377184 | GOTERM_BP_FAT | GO:0016477~cell migration | 11 | 2.39E-04 | CDH13, EDNRB, S100P, AIMP1, CXCR4, ANG, ARID5B, VCAN, THBS1, PPAP2B, CXCL12 |
| 6 | 3.241037 | SP_PIR_KEYWORDS | Growth factor binding | 4 | 2.28E-04 | IGFBP7, IGFBP1, IGFBP3, IGFBP5 |
| 7 | 3.223215 | GOTERM_CC_FAT | GO:0031093~platelet alpha granule lumen | 5 | 4.71E-04 | FGB, CLU, THBS1, THBS2, TIMP1 |
| 8 | 3.144168 | GOTERM_BP_FAT | GO:0055066~di-, tri-valent inorganic cation homeostasis | 11 | 7.37E-05 | EDNRB, CD55, TRPM8, TRPC6, CXCR4, CXCL13, IL6ST, TGM2, MT1H, CXCL12, SOD2 |
| 9 | 3.033787 | UP_SEQ_FEATURE | repeat:TSP type-3 8 | 3 | 5.79E-04 | COMP, THBS1, THBS2 |
| 10 | 2.988334 | GOTERM_BP_FAT | GO:0002541~activation of plasma proteins involved in acute inflammatory response | 6 | 4.52E-05 | CD55, F3, CLU, C1R, C1S, C4BPA |
| 11 | 2.896016 | SP_PIR_KEYWORDS | sushi | 5 | 8.83E-04 | CD55, C1R, VCAN, C1S, C4BPA |
| 12 | 2.795222 | GOTERM_BP_FAT | GO:0030334~regulation of cell migration | 8 | 9.72E-04 | CDH13, CXCR4, IL6ST, F3, THBS1, IGFBP3, CXCL12, IGFBP5 |
| 13 | 2.760289 | SP_PIR_KEYWORDS | metalloproteinase inhibitor | 3 | 1.73E-04 | TIMP2, TIMP3, TIMP1 |
| 14 | 2.752983 | GOTERM_BP_FAT | GO:0043066~negative regulation of apoptosis | 11 | 0.001637 | CDH13, EDNRB, F3, COMP, CLU, BCL2A1, TGM2, FOXO1, THBS1, ANXA4, SOD2 |
| 15 | 2.729432 | SP_PIR_KEYWORDS | complement pathway | 5 | 6.70E-05 | CD55, CLU, C1R, C1S, C4BPA |
| 16 | 2.692459 | INTERPRO | IPR002345:Lipocalin | 4 | 0.001215 | LCN2, RBP4, APOD, PAEP |
| 17 | 2.454405 | SP_PIR_KEYWORDS | chelation | 3 | 0.001576 | MT1E, MT1H, MT1G |
| 18 | 2.413648 | GOTERM_BP_FAT | GO:0050921~positive regulation of chemotaxis | 4 | 0.002361 | CDH13, F3, THBS1, CXCL12 |
| 19 | 2.321079 | GOTERM_BP_FAT | GO:0042325~regulation of phosphorylation | 12 | 0.003916 | LIF, EDNRB, MAP3K5, CXCR4, ANG, IL6ST, ADRA2A, SFN, THBS1, IGFBP3, GADD45A, DUSP6 |
| 20 | 2.23256 | INTERPRO | IPR009168:Insulin-like growth factor binding protein | 3 | 0.001058 | IGFBP1, IGFBP3, IGFBP5 |
| 21 | 2.172174 | COG_ONTOLOGY | Cytoskeleton | 4 | 5.51E-04 | TAGLN, PLS1, CNN1, LCP1 |
| 22 | 2.111655 | GOTERM_BP_FAT | GO:0042981~regulation of apoptosis | 16 | 0.007217 | BCL2A1, CLU, FOXO1, SFN, ANXA4, TIMP3, SOD2, EDNRB, CDH13, MAP3K5, NUPR1, COMP, F3, TGM2, THBS1, IGFBP3 |
| 23 | 1.937672 | GOTERM_BP_FAT | GO:0030335~positive regulation of cell migration | 5 | 0.009273 | CDH13, IL6ST, F3, THBS1, CXCL12 |
| 24 | 1.875423 | GOTERM_BP_FAT | GO:0042327~positive regulation of phosphorylation | 5 | 0.01244 | LIF, EDNRB, ANG, IL6ST, THBS1 |
| 25 | 1.827148 | UP_SEQ_FEATURE | domain:TSP N-terminal | 3 | 0.013372 | COL15A1, THBS1, THBS2 |
| 26 | 1.804195 | GOTERM_BP_FAT | GO:0007596~blood coagulation | 5 | 0.014733 | CD36, THBD, FGB, F3, PAPSS2 |
| 27 | 1.619356 | GOTERM_MF_FAT | GO:0030169~low-density lipoprotein binding | 3 | 0.014394 | CDH13, CD36, THBS1 |
| 28 | 1.555537 | INTERPRO | IPR018048:Small chemokine, C-X-C, conserved site | 3 | 0.009023 | CXCL14, CXCL13, CXCL12 |
| 29 | 1.358717 | GOTERM_BP_FAT | GO:0006461~protein complex assembly | 11 | 0.018578 | TRPM8, ANG, FGB, GPX3, MYH11, TGM2, CDA, GJA1, PPARGC1A, SLC1A1, SOD2 |
| 30 | 1.317655 | GOTERM_BP_FAT | GO:0045859~regulation of protein kinase activity | 8 | 0.040944 | MAP3K5, CXCR4, ANG, ADRA2A, SFN, THBS1, GADD45A, DUSP6 |
| 31 | 1.296324 | SP_PIR_KEYWORDS | Serine protease | 5 | 0.020559 | FAP, C1R, C1S, DPP4, HABP2 |
| 32 | 1.226636 | GOTERM_BP_FAT | GO:0042098~T cell proliferation | 3 | 0.025571 | CXCR4, IL15, CXCL12 |
| 33 | 1.070548 | GOTERM_BP_FAT | GO:0042110~T cell activation | 5 | 0.029356 | CXCR4, IL15, CXCL12, DPP4, LCP1 |
