# Supplementary material for: Endometrial Receptivity: A Revisit to Functional Genomics Studies on Human Endometrium and Creation of HGEx-ERdb
Source: PLoS One. 2013 Mar 26;8(3):e58419. doi: 10.1371/journal.pone.0058419 (PMC3608645; doi:10.1371/journal.pone.0058419)
Supplement: Table S3 — Functional annotation of Down-Rep Down-Nd RAGs using DAVID software. (DOCX) [file pone.0058419.s006.docx]

**Table S3: Functional annotation of Down-Nd RAGs using DAVID software (p<0.05)**

| **Annotation Cluster Number** | **Enrichment Score** | **Category** | **Term** | **Count** | **PValue** | **Genes** |
| --- | --- | --- | --- | --- | --- | --- |
| 1 | 11.8186 | UP_SEQ_FEATURE | calcium-binding region:2; high affinity | 7 | 1.82E-13 | S100A5, S100B, S100A7, S100A7A, S100G, S100Z, S100A2 |
| 2 | 2.238177 | UP_SEQ_FEATURE | domain:EF-hand 1 | 8 | 3.57E-09 | S100A5, S100B, S100A7, S100A7A, S100G, S100Z, CALB2, S100A2 |
| 3 | 2.238177 | GOTERM_BP_FAT | GO:0007409~axonogenesis | 4 | 0.002341 | S100B, EFNA2, EPHB3, BMP7 |
| 4 | 1.394355 | SP_PIR_KEYWORDS | mitosis | 3 | 0.025189 | CENPE, CDC20, PTTG1 |
| 5 | 0.704844 | GOTERM_BP_FAT | GO:0006366~transcription from RNA polymerase II promoter | 3 | 0.041994 | E2F2, FOXM1, PTTG1 |
